# Characterization of anti-MERS-CoV antibodies against various recombinant structural antigens of MERS-CoV in an imported case in China

**DOI:** 10.1038/emi.2016.114

**Published:** 2016-11-09

**Authors:** Wenling Wang, Huijuan Wang, Yao Deng, Tie Song, Jiaming Lan, Guizhen Wu, Changwen Ke, Wenjie Tan

**Affiliations:** 1 Key Laboratory of Medical Virology, Ministry of Health, National Institute for Viral Disease Control and Prevention, Chinese Center for Disease Control and Prevention, Beijing 102206, China; 2 Guangdong Provincial Center for Disease Control and Prevention, Guangzhou 511430, Guangdong Province, China

**Keywords:** antibody, enzyme-linked immunosorbent assay, MERS-CoV, patient, recombinant structural antigens

## Abstract

The first imported case of Middle East respiratory syndrome (MERS) in China recently occurred, allowing for the characterization of antibody titers in a series of the patient's sera using the following methods based on recombinant viral structural antigens: inactivated MERS coronavirus (MERS-CoV) enzyme-linked immunosorbent assay (ELISA), recombinant MERS-CoV spike (S, or fragments of S) ELISA, nucleoprotein (NP) ELISA and MERS S pseudovirus particle-based neutralization test (ppNT). A longitudinal profile of the infection showed that seroconversion detected by ELISAs based on the recombinant extracellular domain, S, S1 and receptor-binding domain (RBD) antigens occurred as early as neutralizing antibodies were detected by the ppNT and earlier than antibodies were detected by the inactivated MERS-CoV and N-terminal domain (NTD) ELISAs. Antibodies detected by the NP ELISA occurred last. Strong correlations were found between the S1, RBD and NP ELISAs and the inactivated MERS-CoV ELISA. The S and RBD ELISAs were highly correlated with the commercial S1 ELISA. The S ELISA strongly correlated with the ppNT, although the MERS-CoV, S1, NTD and RBD ELISAs were also significantly correlated with the ppNT (*P*<0.001).

## Introduction

Middle East respiratory syndrome (MERS) is caused by MERS coronavirus (MERS-CoV), and as of 23 March 2016, it had affected 26 countries, with 1698 cases and at least 609 deaths.^1^ Sensitive serological assays independent of the use of live MERS-CoV and biosafety level 3 laboratories are important for clinical diagnosis and research. Recombinant antigen-based enzyme-linked immunosorbent assays (ELISAs) are preferred for diagnosis owing to their high efficiency and reproducibility. Importantly, such assays do not require cultivation of MERS-CoV in biosafety level 3 facilities, which are not readily available in most laboratories. Although an S1-based ELISA Kit is commercially available and has been used in many studies,^2, 3, 4, 5, 6^ data are limited on comparing ELISAs that detect MERS-CoV antibodies in humans based on alternative recombinant antigens, including the extracellular domain (representative of the full-length spike protein (S)), N-terminal domain (NTD), receptor-binding domain (RBD) and nucleoprotein (NP). We used a series of sera from the first imported case of MERS in China to longitudinally evaluate antibody production and to compare the antigenicity of whole inactivated MERS-CoV, S, S1, NTD, RBD and NP. The sera were also used to investigate the correlation of ELISAs with the pseudovirus particle-based neutralization test (ppNT).

## Materials and Methods

### Case report and blood samples

The first MERS-CoV case in China was imported from the Republic of Korea and confirmed during hospital admission at the end of May 2015. A case investigation revealed a history of exposure to the first confirmed MERS case in South Korea when the male patient in question had visited his father who had been admitted to the same hospital ward as that of the Korean patient with MERS-CoV. On 20 May, while in Korea, the patient complained of feeling unwell but had no respiratory symptoms; his temperature was 38.7 °C on 25 May. However, 1 day later, against medical advice, he traveled from South Korea to Guangdong province (mainland China) via Hong Kong. On 27 May 2015, the Chinese Ministry of Health was notified of the patient's entry into China by the World Health Organization (WHO). The man was confirmed to be MERS-CoV-positive on 28 May and was immediately admitted to a hospital.^7^ He was discharged from the hospital at the end of June 2015. A series of venous blood samples were collected from the patient after he was admitted to the hospital. Normal control sera from 40 healthy blood donors (both sexes) were used to detect background values and calculate the cutoff values for the methods used in this study. The blood samples were processed within 24 h of collection, and the sera were stored at −80 °C.

### Virus preparation

MERS-CoV (strain EMC/2012) was kindly provided by Professor Ron Fouchier (Erasmus Medical Centre, Rotterdam, The Netherlands). The virus was propagated in Vero cells (ATCC, Manassas, VA, USA) in Dulbecco's modified Eagle medium supplemented with 2% fetal calf serum, 100 international units/mL penicillin and 100 μg/mL streptomycin at 37 °C in 5% CO_2_. All experiments related to live MERS-CoV were performed according to the standard operating procedures of the biosafety level-3 facility in the Chinese Center for Disease Control and Prevention (China CDC). Quantified MERS-CoV was inactivated with 0.4% formaldehyde for seven days. The inactivated MERS-CoV was then centrifuged at 3000 r.p.m. for 1 min at 4 °C to collect the supernatant after confirmation that it was non-infectious by titration in Vero cells. Subsequently, the supernatant was concentrated by centrifugation at 24 000 r.p.m. for 2 h at 4 °C, and the pellet was collected and dissolved in phosphate-buffered saline (PBS) overnight. The concentrated MERS-CoV was quantified using a BCA-Based Protein Quantification Kit (Applygen Technologies Inc., Beijing, China) and stored at −80 °C until use. In addition, the inactivated MERS-CoV was characterized by sodium dodecyl sulfate-polyacrylamide gel electrophoresis and western blotting (WB) assay.

### Preparation of recombinant proteins

The recombinant His-tagged extracellular domain (amino acids 1–1297) of MERS-CoV (HCoV-EMC/2012) spike (S) expressed in Baculovirus–Insect cells was purchased from Sino Biological Inc. (Beijing, China). Construction of expression plasmids for the RBD (amino acids 367–606) of MERS-CoV and the method used for subsequent expression in Baculovirus–Insect cells were described previously.^8^ The NTD (amino acids 18–353) of S was expressed in Baculovirus–Insect cells and was provided by the Institute of Microbiology, Chinese Academy of Sciences. Expression and purification of NP in a prokaryotic system were performed as described previously.^9^ Purified NTD, RBD and NP were identified by sodium dodecyl sulfate-polyacrylamide gel electrophoresis and WB.

### Serological tests

ELISA plates were coated at 4 °C overnight with inactivated MERS-CoV particles or recombinant proteins (50 ng/well) in carbonate buffer (pH 9.6). Each well of the plates was then incubated with blocking solution consisting of 10% goat serum in PBS for two h at 37 °C. The wells were washed five times with PBS containing 0.05% Tween 20 (PBS-T). Aliquots of 100 μL of serially diluted sera were added to wells of the ELISA plates, followed by further incubation for 1.5 h at 37 °C. After five washes with PBS-T, the plates were incubated with horseradish peroxidase (HRP)-labeled goat anti-human immunoglobulin G (IgG) or IgM antibodies for one h at 37 °C. 3,3',5,5'-tetramethylbenzidine (Sigma, St. Louis, MO, USA) was added at 100 μL/well after five washes with PBS-T, and the wells were incubated for 5 min at room temperature. Then, 50 μL of 2 M H_2_SO_4_ was added to each well to terminate the reaction, and the optical density (OD) was immediately read at 450 nm. The average OD_450_ values for the normal controls were calculated for IgG and IgM, and the cutoff values were determined as the average of normal controls+3 SD. The same normal control sera mixed in equal volumes served as negative controls.

The S1-based ELISA Kit for detecting IgG was purchased from EUROIMMUNE (Luebeck, Germany), and analyses were performed according to the manufacturer's instructions. The kit included a calibrator for defining the cutoff value recommended by the manufacturer. Values below the cutoff were considered negative and those above were considered positive. S1-specific IgM levels were not determined using an S1-based ELISA Kit because such determinations were outside the range of applications of the kit.

Pseudovirus expressing the MERS-CoV S protein was prepared using the Env-defective and luciferase-expressing HIV-1 genome, and the ppNT was performed as described previously.^10^ Briefly, a lentivirus-based MERS-CoV S pseudovirus was preincubated with serially diluted serum samples from the patients with MERS-CoV at 37 °C for one h, and the mixtures were distributed into 96-well plates containing monolayers of Huh7.5 cells. After 24 h of incubation, fresh medium was added, followed by incubation for an additional 48 h. The cells were washed with PBS and then lysed using the lysis reagent included in the Luciferase Kit (Promega, Madison, WI, USA). Aliquots of cell lysates were transferred to 96-well Costar flat-bottomed luminometry plates (Corning Costar, Corning, NY, USA), followed by addition of the luciferase substrate (Promega). Relative fluorescence intensity values were immediately determined using a Gaomax luminometer (Promega). The luciferase activity of Huh7.5-CD81 cells treated with pseudovirus alone (reference group) was defined as 100% infection. Cells not treated with pseudovirus were included as a background control. The results were expressed as the percentage of infection compared with those of the control group (Huh7.5 cells treated with only pseudovirus preparations). Fifty percent reductions in relative fluorescence intensity were used to calculate MERS-CoV-specific neutralizing antibody (NAb) titers in the sera. The NAb titers were defined as the highest serum dilutions that resulted in a 50% reduction in luciferase activity. NAb titers lower than 1:100 were considered negative.

### Statistical analysis

All experiments were performed at least three times. All data were analyzed using SPSS (IBM, New York, NY, USA) and Graphpad Prism 5 (Graphpad Software Inc., La Jolla, CA, USA). Pearson's correlation coefficients between different assays were calculated, and significance was defined as *P*<0.05.

## Results

Inactivated MERS-CoV was characterized by WB and was shown to contain S and NP viral components (Supplementary Figure S1). Purified NTD, RBD and NP were found to be highly pure and antigenic by WB (Supplementary Figure S1). Normal controls, i.e., sera from 40 healthy blood donors, were used to determine background values and calculate cutoff levels. IgG and IgM against inactivated MERS-CoV, S, NTD, RBD and NP and IgG against S1 in the control sera were detected by ELISA, and cutoff values were determined (Supplementary Figure S2).

**Figure 1:**
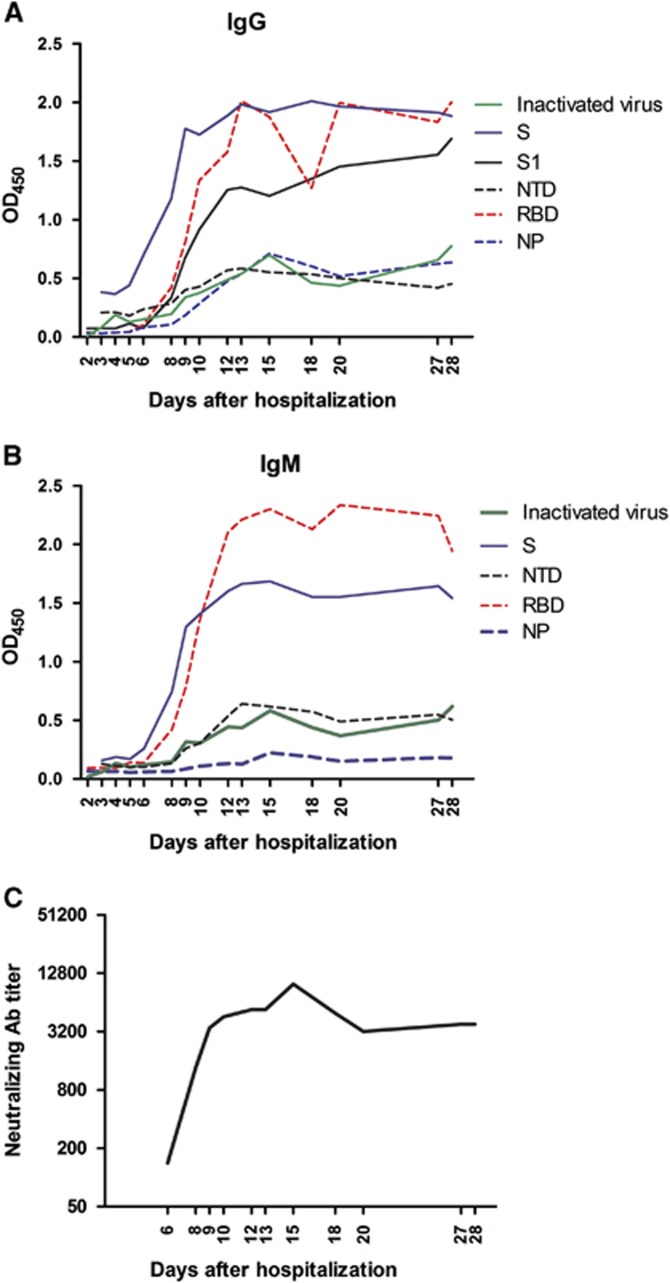
Kinetics of the serological response in a MERS-CoV-infected patient as determined by ELISAs for various recombinant antigens and the ppNT. Sera sampled on days 2, 3, 4, 5, 6, 8, 9, 10, 12, 13, 15, 18, 20, 27 and 28 after admission to the hospital were retrospectively analyzed. (**A**) Longitudinal profiles of IgG antibodies against MERS-CoV, S, S1, NTD, RBD and NP in the patient who was the first to import MERS-CoV into China; sera were determined by 1: 80 dilution in all ELISAs except by 1:101 dilution in S1 ELISA. (**B**) Longitudinal profiles of IgM antibodies against MERS-CoV, S, NTD, RBD and NP; sera were determined by 1:80 dilutioin in all ELISAs. (**C**) Longitudinal profiles of NAb against MERS-CoV. enzyme-linked immunosorbent assay, ELISA; immunoglobulin G/M, IgG/M; Middle East respiratory syndrome coronavirus, MERS-CoV; neutralizing antibody, NAb; nucleoprotein, NP; N-terminal domain, NTD; optical density, OD; pseudovirus particle-based neutralization test, ppNT; receptor-binding domain, RBD; recombinant MERS-CoV full-length spike protein, S.

**Figure 2:**
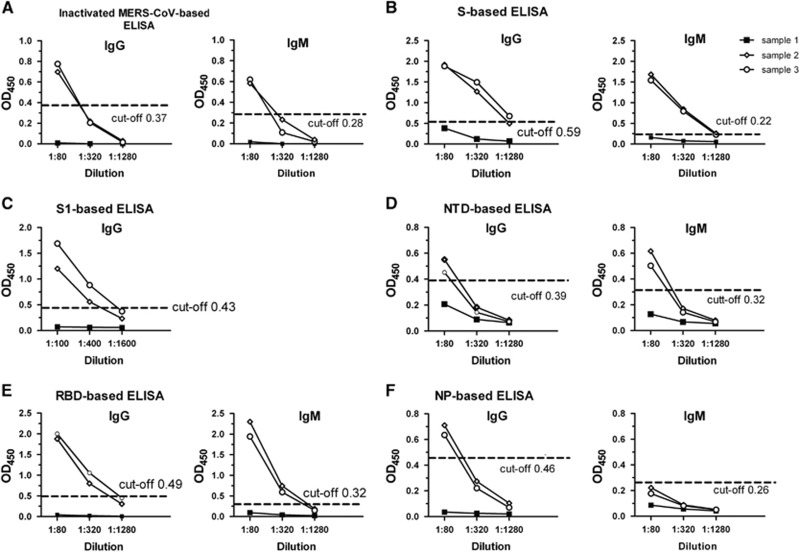
Sensitivities of ELISAs based on different viral antigens. ELISA plates were coated with inactivated MERS-CoV particles (**A**) or purified recombinant S (**B**), S1 (**C**), NTD (**D**), RBD (**E**) or NP (**F**) protein. Serum samples from the patient with MERS-CoV collected on days 2 (sample 1), 15 (sample 2) and 28 (sample 3) after admission to the hospital were serially diluted and dispensed into the wells of an ELISA plate. HRP-labeled goat anti-human IgG (left) and IgM (right) were used as the secondary antibody, with 3,3',5,5'-tetramethylbenzidine (TMB) as the substrate. The results are expressed as the absorbance reading at 450 nm. As the negative control had very poor ELISA responses, the results are not shown to avoid interference with the target profiles. enzyme-linked immunosorbent assay, ELISA; horseradish peroxidase, HRP; immunoglobulin G/M, IgG/M; Middle East respiratory syndrome coronavirus, MERS-CoV; nucleoprotein, NP; N-terminal domain, NTD; optical density, OD; receptor-binding domain, RBD; recombinant MERS-CoV full-length spike protein, S.

A longitudinal profile of binding antibodies (IgG and IgM) and NAb against MERS-CoV in sera from the first case of imported MERS-CoV in China was analyzed depending on the whole MERS-CoV and recombinant protein ELISAs and the ppNT. Figures 1 and 2 showed that the ELISAs readily detected specific antibodies in the patient's sera. The MERS-CoV S-based ELISA showed the highest sensitivity, followed by the S1- and RBD-based ELISAs; the inactivated MERS-CoV-, NTD- and NP-based ELISAs were not as sensitive as the S-, S1- and RBD-based ELISAs. Moreover, the S-, S1- and RBD-based ELISAs showed broader measurement ranges than those based on inactivated MERS-CoV, NTD and NP. In the ppNT assay, NAb positivity appeared as early as anti-S antibody positivity, as determined by the ELISAs (Figure 1). Anti-S antibodies were first detected by ELISA (IgM on day 6 and IgG on day 5 after admission), followed by antibodies against S1 and RBD, then antibodies against MERS-CoV particles and NTD and, finally, antibodies against NP (by days 12–13 after admission) (Supplementary Figure S3).

**Figure 3:**
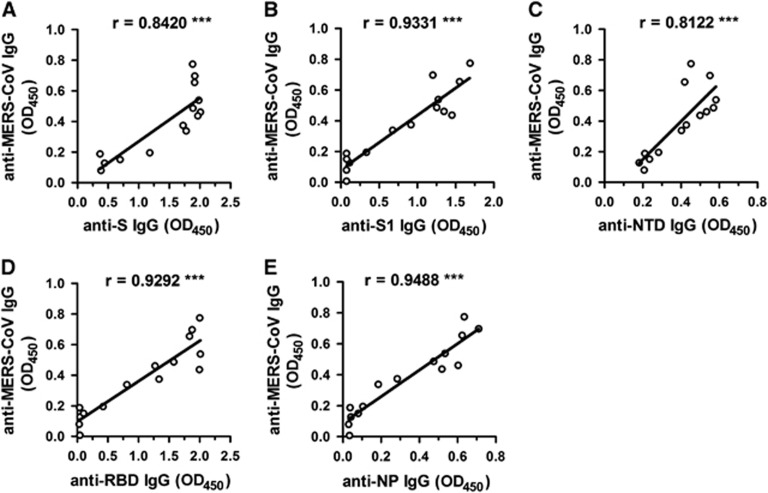
Correlations among the inactivated virus-based and other S or N protein-based ELISA results. The absorbance readings of the S (**A**), S1 (**B**), NTD (**C**), RBD (**D**) and NP (**E**) based IgG ELISAs (OD_450_ values) were plotted against that of the virus-based IgG ELISA (OD_450_ values) (data are those from Figure 1A). ***P<0.001. Enzyme-linked immunosorbent assay, ELISA; immunoglobulin G, IgG; Middle East respiratory syndrome coronavirus, MERS-CoV; nucleoprotein, NP; N-terminal domain, NTD; optical density, OD; receptor-binding domain, RBD; recombinant MERS-CoV full-length spike protein, S.

**Figure 4:**
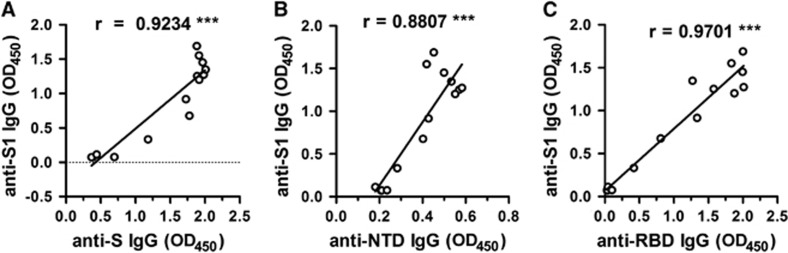
Correlation of the Euroimmune EIA Kit for detecting anti-S1 IgG antibody of MERS-CoV (OD_450_ values) with other recombinant protein-based IgG ELISAs (OD_450_ values) used in this study. The absorbance readings of the S (**A**), NTD (**B**) and RBD (**C**) based IgG ELISAs (OD_450_ values) were plotted against those of the S1-based IgG ELISA (OD_450_ values) (data are those from Figure 1A). ***P<0.001. Enzyme-linked immunosorbent assay, ELISA; immunoglobulin G, IgG; Middle East respiratory syndrome coronavirus, MERS-CoV; nucleoprotein, NP; N-terminal domain, NTD; optical density, OD; receptor-binding domain, RBD; recombinant MERS-CoV full-length spike protein, S.

The whole virus-based ELISA kit was able to detect antibodies specific for all structural components of the virus. In the present study, correlations of the recombinant protein-based ELISAs with that based on inactivated MERS-CoV were analyzed. The scatter plots in Figure 3 show excellent correlations between the MERS-CoV ELISA and the S1, RBD and NP ELISA, with Pearson's correlation coefficients of 0.9292–0.9488. S and NTD ELISAs were less strongly correlated with the MERS-CoV ELISA, with Pearson's correlation coefficients of 0.8122–0.8420. Subsequently, correlations of the S, NTD and RBD ELISA results with those of the commercial S1 ELISA were examined. The scatter plots in Figure 4 show excellent correlations between the S1 ELISA and the S and RBD ELISAs, with Pearson's correlation coefficients of 0.9234–0.9701, and the NTD ELISA was less strongly correlated with the S1 ELISA, with a Pearson's correlation coefficient of 0.8807.

**Figure 5:**
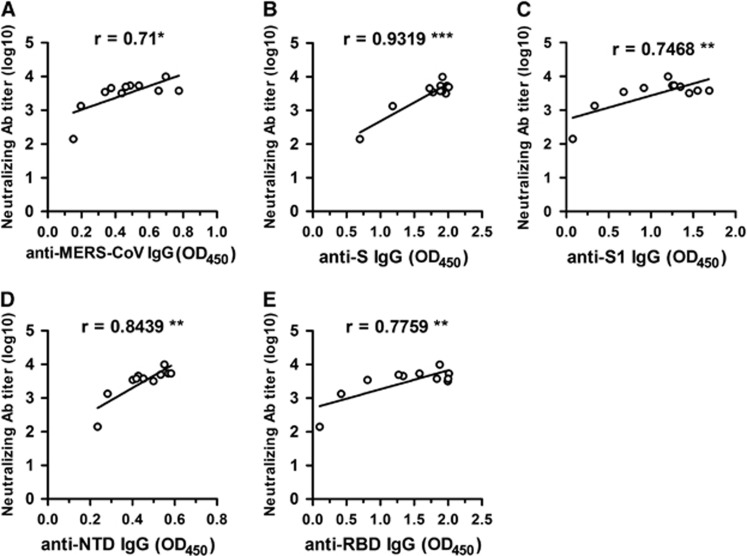
Correlations among the ELISAs (IgG) (OD_450_ values) and the MERS-CoV ppNT assay (neutralizing antibody titer) used in this study. The absorbance readings of the inactivated MERS-CoV (**A**), S (**B**), S1 (**C**), NTD (**D**) and RBD (**E**) based IgG ELISAs (OD_450_ values) (data are those from Figure 1A) were plotted against neutralizing antibody titers (data are those from Figure 1C). *P<0.05; **P<0.01; ***P<0.001. Antibody, Ab; enzyme-linked immunosorbent assay, ELISA; immunoglobulin G, IgG; Middle East respiratory syndrome coronavirus, MERS-CoV; nucleoprotein, NP; N-terminal domain, NTD; optical density, OD; pseudovirus particle-based neutralization test, ppNT; receptor-binding domain, RBD; recombinant MERS-CoV full-length spike protein, S.

The MERS S ppNT is a sensitive and specific assay used for detecting NAbs against MERS-CoV. Here we examined the relationship between the ppNT and various ELISAs. The scatter plots in Figure 5 show that only the S ELISA had a strong correlation with the ppNT, with a Pearson's correlation coefficient of 0.9319. The MERS-CoV, S1, NTD and RBD ELISAs were less strongly correlated with the MERS S ppNT, with Pearson's correlation coefficients of 0.71–0.8439.

## Discussion

To the best of our knowledge, this is the first comprehensive investigation of the anti-MERS-CoV antibody profile in sera from a MERS patient based on a multiplex analysis of antibodies against both the S protein and NP. The longitudinal profiles of IgG and IgM antibodies in the sera from this case, the first to be imported into China, showed that seroconversion of antibodies detected by the S, S1 and RBD ELISAs occurred earlier than that detected by the inactivated MERS-CoV ELISA. The results shown in Figures 1 and 2 further illustrate that the S, S1 and RBD ELISAs are more sensitive than the whole MERS-CoV ELISA in detecting specific antibodies. In a previous severe acute respiratory syndrome coronavirus (SARS-CoV) study, an S protein-based ELISA also appeared to be more sensitive than a virus-based Huada ELISA for detecting SARS-CoV-specific antibodies.^11^ The NP of SARS-CoV is an immunodominant antigen that is used to detect SARS-CoV infection.^11, 12^ In the present study, our NP-based ELISA illustrated the benefit of using recombinant NP for detecting MERS-CoV infection. However, seroconversion of NP-specific antibodies occurred later than seroconversion of antibodies against several S proteins and inactivated MERS-CoV. Woo *et al.*^12^ evaluated the longitudinal profile of IgG and IgM antibodies against SARS-CoV NP in patients with pneumonia due to SARS-CoV. The study showed that the levels of the two antibodies increased to detectable levels by the third week of illness, which was approximately 1 week later than that observed in another study by Lie *et al.*,^13^ based on a whole SARS-CoV ELISA. The 1-week delay in seroconversion of NP-specific antibodies in the study by Woo *et al.*^12^ compared with the results of Li *et al.*^13^ may reflect reduced sensitivity of NP-based ELISAs based on inactivated virus particles for detecting SARS-CoV infection at an early stage of illness, although clinical treatment may also interfere with the appearance of antibodies. The results of the two studies confirmed the characteristics of NP-specific antibodies in different contexts. Woo *et al.*^12^ reported that IgM antibodies against SARS-CoV NP increased to detectable levels later than IgG antibodies, in contrast to the phenomena described for most other pathogens. We found that IgM antibodies against some antigens were detectable at the same time or later than IgG in the present study, which may have been due to the earlier development of IgG than IgM antibodies or the different sensitivities of the Ig class-specific ELISAs.^12^

The S1-based ELISA Kit has the advantages of easy operation and convenience, and it has been validated and applied in many studies.^2, 3, 4, 5, 6, 14, 15^ In the present study, gradual increases in S1-specific serum IgG antibody levels were observed in the first patient to import MERS-CoV into China, as was the case for most MERS-CoV patients in other studies performed in South Korea^6, 15^ and the Kingdom of Saudi Arabia.^14^ Seroconversion of the S1-specific IgG antibody was observed 8 days after admission (16 days after the onset of illness), which was consistent with previous findings.^6^ Moreover, the S1 ELISA maintained an excellent correlation with inactivated MERS-CoV and S ELISAs (*P*<0.001). In the present study, the recombinant RBD (which is the main epitope of S1) was as sensitive as S1 for detecting MERS-CoV infection, and there was an excellent correlation between the RBD ELISA and S1 ELISA, with a Pearson's correlation coefficient of 0.9701. Therefore, RBD can be developed as an alternative antigen for ELISA kits to detect MERS-CoV infection.

The MERS-spike ppNT assay is preferred owing to its lack of a requirement for biosafety level 3 facilities, and it has been used extensively and validated for seroepidemiology in humans, with a good correlation between the MERS ppNT and PRNT90.^3^ The present study confirmed the sensitivity of the ppNT when it was used to detect NAb at an early stage of illness. Moreover, the ppNT assay showed an excellent correlation with the S ELISA, although the latter is a binding assay while the former is a functional neutralizing assay. The MERS-CoV, S1, NTD and RBD ELISAs (OD_450_) had acceptable but lower Pearson's correlation coefficients (0.71–0.8439) when compared with the ppNT titer. We speculate that this is because there are multiple components irrelevant to neutralization in inactivated MERS-CoV and fewer epitopes related to neutralization in recombinant S1, NTD and RBD.

Our study has certain limitations. We studied only one patient who was followed for only one month after admission to the hospital. Although only one MERS-CoV case has been reported in China, additional cases with longer follow-ups are needed to comprehensively understand the MERS-CoV-derived antibody profile and to verify the specificity and sensitivity of MERS-CoV- and recombinant protein-based ELISAs. Fortunately, a series of serum samples was collected from this case. Additionally, a commercial S1-based ELISA Kit was available and was adopted as the reference test. We found that the ELISAs of various recombinant structural proteins, including S, S1, NTD, RBD and NP, as well as the ppNT assay, can be used for MERS-CoV diagnosis and seroepidemiology. The S, S1 and RBD ELISAs were more sensitive than other ELISA formats, especially the S1 and RBD ELISAs were advantageous because the recombinant S1 and RBD are smaller than the full-length S and easier to express and purify.

In conclusion, an RBD ELISA may be an alternative to an S1 ELISA for determining infection at an early stage and in patients with poor serological responses to MERS-CoV. In addition, an S ELISA may be an useful alternative to the MERS S ppNT, which was shown to be a reliable surrogate of neutralization activity, and may be useful for large-scale seroepidemiological studies of MERS-CoV infection.
