# Supplementary material for: Characterization of anti-MERS-CoV antibodies against various recombinant structural antigens of MERS-CoV in an imported case in China
Source: Emerg Microbes Infect. 2016 Nov 9;5(11):e113–. doi: 10.1038/emi.2016.114 (PMC5148018; doi:10.1038/emi.2016.114)
Supplement: Supplementary Figure S1 [file emi2016114x1.pdf]

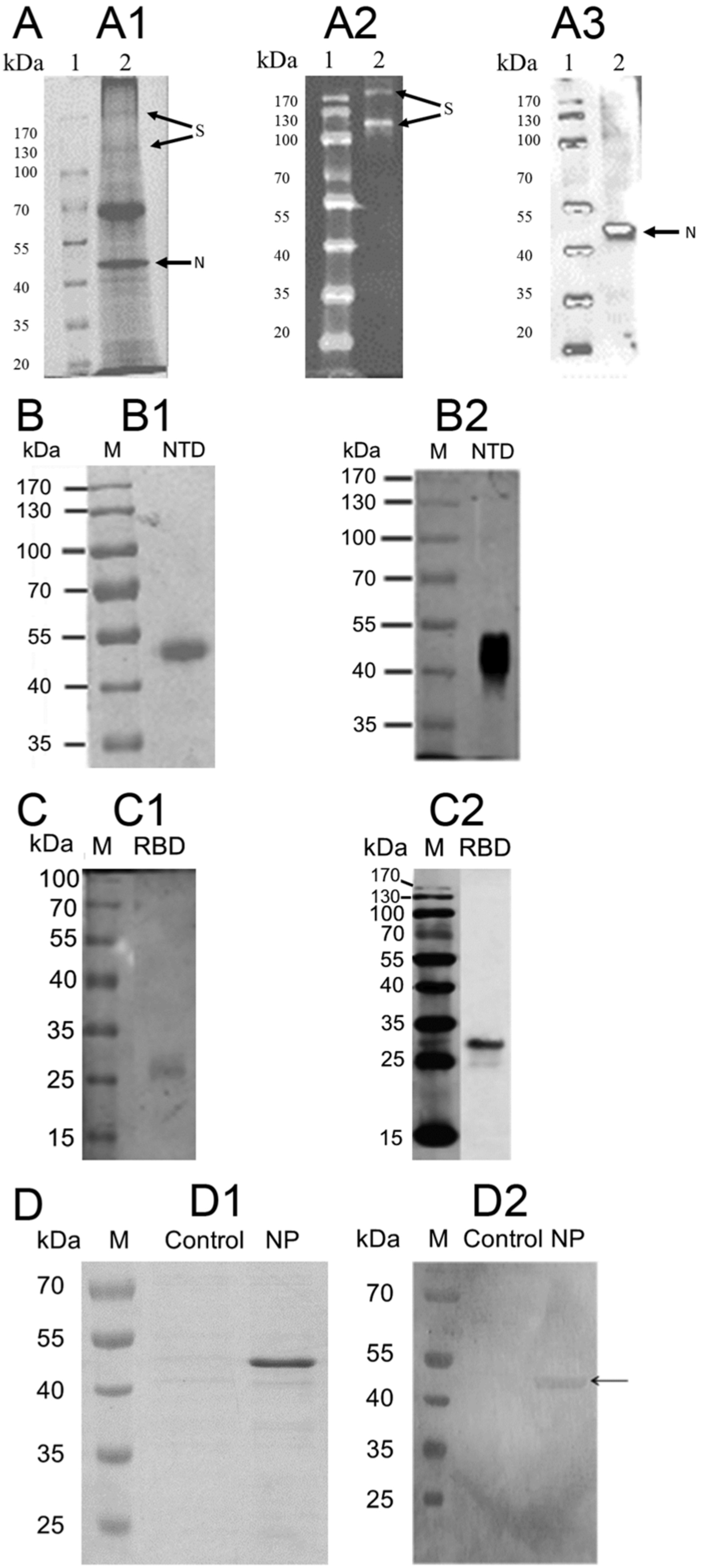

Supplementary Figure S1 Sodium dodecyl sulfate-polyacrylamide gel electrophoresis (SDS-PAGE) and Western blotting (WB) analysis of various antigens used for enzyme linked immunosorbent assay (ELISA) in this study. Inactivated MERS-CoV (A), NTD (B), RBD (C), and NP (D) were run on two or three identical SDS-PAG-Es. One of the gels was stained with coomassie blue (A1, B1, C1, and D1). The protein bands in the unstained gels were transferred onto a nitrocellulose membrane for WB using specific antibodies (A2, mouse anti-S MAb; A3, rabbit anti-NP polyclonal Ab (PcAb); B2, mouse anti-NTD PcAb; C2 mouse anti-MERS-CoV PcAb; and D2, rabbit anti-KLH-NP389-404 PcAb). The second detecting antibody was infrared luciferase-labeled goat-anti-mouse IgG in A2; infrared luciferase-labeled goat-anti-rabbit IgG in A3 and B2; HRP-labeled goat-anti-mouse IgG in C2; HRP-labeled goat-anti-rabbit IgG in D2. M, protein molecular weight markers.
