# Supplementary material for: Characterization of anti-MERS-CoV antibodies against various recombinant structural antigens of MERS-CoV in an imported case in China
Source: Emerg Microbes Infect. 2016 Nov 9;5(11):e113–. doi: 10.1038/emi.2016.114 (PMC5148018; doi:10.1038/emi.2016.114)
Supplement: Supplementary Figure S2 [file emi2016114x2.pdf]

Parameter	Inactivated MERS-CoV		S		S1	NTD		RBD		NP	
	IgG	IgM	IgG	IgM	IgG	IgG	IgM	IgG	IgM	IgG	IgM
X	0.20	0.15	0.27	0.12	0.10	0.21	0.16	0.26	0.18	0.24	0.15
STD	0.06	0.04	0.11	0.03	0.05	0.06	0.05	0.08	0.05	0.07	0.04
X+3×STD	0.37	0.28	0.59	0.22	0.26	0.39	0.32	0.49	0.32	0.46	0.26

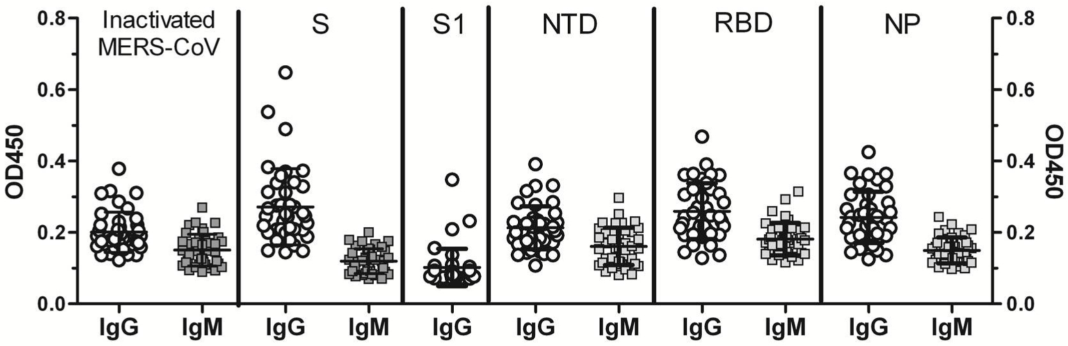

Supplementary Figure S2 Determination of cut-off values for the different ELISAs evaluated in the detection of IgG or IgM antibodies in patients. Sera from 40 healthy adult donors were diluted 1:80 for antibody detection, with the exceptions of 1:101 for anti-S1 IgG detection. IgG and IgM were evaluated using the inactivated MERS-CoV virion-, S-, S1-, NTD-, RBD-, and NP-based ELISAs. HRP-labeled goat anti-human IgM or IgG was used as the secondary antibody, with 3,3',5,5'-tetramethylbenzidine used as the substrate, and the absorbance was determined at 450 nm. The cut-off values were calculated as the mean absorbance readings + 3 standard deviations of the serum samples from all 40 blood donors. *, Cut-off value for S1-based ELISA determined based on the calibrator supplied with the commercial kit.
