# Supplementary material for: Characterization of anti-MERS-CoV antibodies against various recombinant structural antigens of MERS-CoV in an imported case in China
Source: Emerg Microbes Infect. 2016 Nov 9;5(11):e113–. doi: 10.1038/emi.2016.114 (PMC5148018; doi:10.1038/emi.2016.114)
Supplement: Supplementary Figure S3 [file emi2016114x3.pdf]

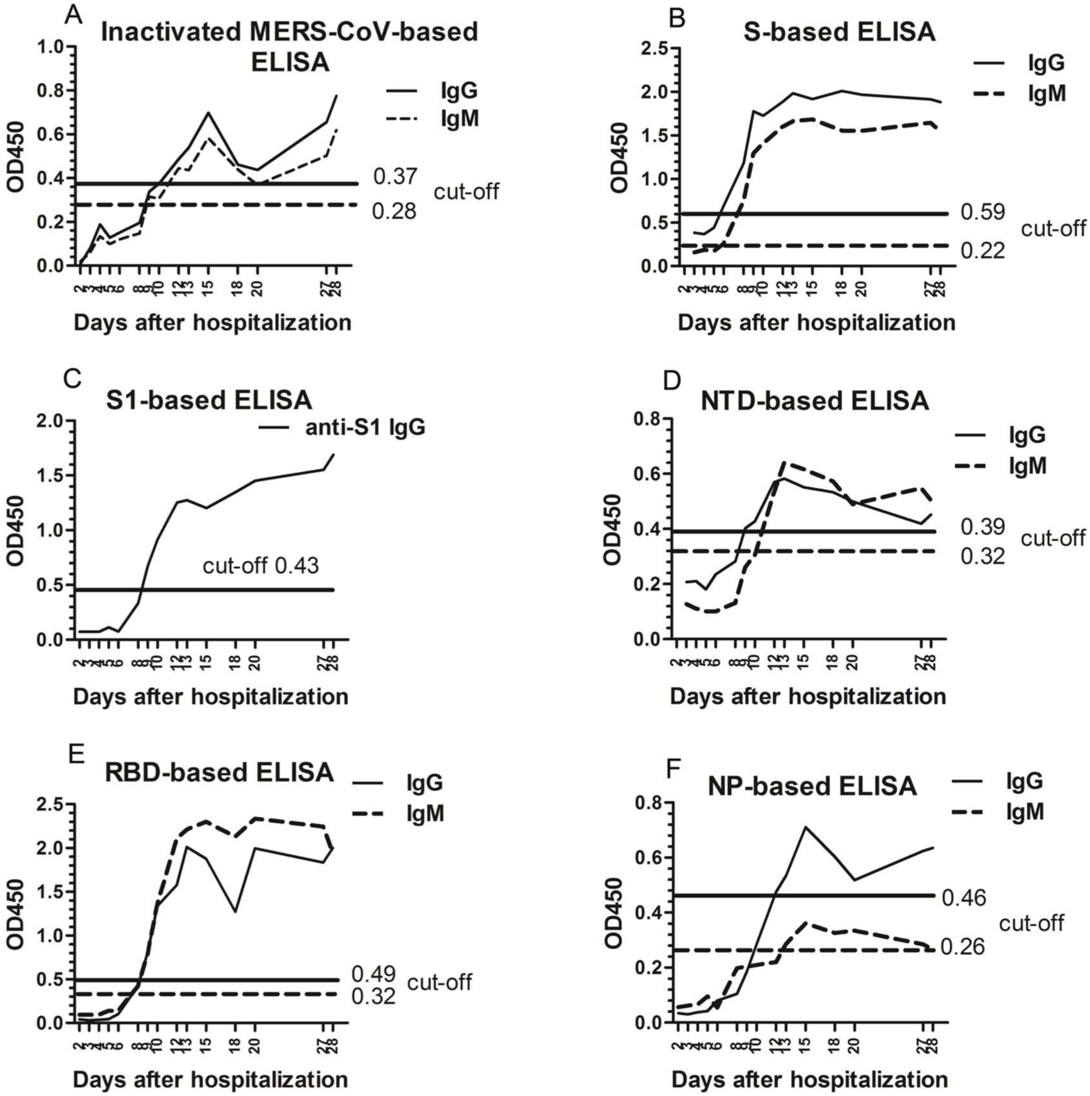

Supplementary Figure S3 Kinetics of IgG and IgM responses in the first imported MERS-CoV patient in China using various ELISA with different antigens. A-F, ELISA plates were coated with inactivated MERS-CoV particles (A), purified S (B), S1 (C), NTD (D), RBD (E) and NP (F) protein respectively. Serum samples obtained from the imported MERS-CoV patient after admission were diluted 1:80 (A, B, D, E and F) or 1:101 (C), and the results were expressed as the absorbance readings at 450 nm.
